# Die Einstellung der deutschen Bevölkerung zu psychischen Störungen

**DOI:** 10.1007/s00103-023-03679-3

**Published:** 2023-02-28

**Authors:** Georg Schomerus, Jenny Spahlholz, Sven Speerforck

**Affiliations:** 1 Klinik und Poliklinik für Psychiatrie und Psychotherapie, Universitätsklinikum Leipzig– AöR, Semmelweisstr. 10, 04103 Leipzig, Deutschland; 2 Medizinische Fakultät, Klinik und Poliklinik für Psychiatrie und Psychotherapie, Universität Leipzig, Leipzig, Deutschland

**Keywords:** Stigma, Trendstudien, Repräsentative Bevölkerungsbefragung, Depression, Schizophrenie, Stigma, Trend studies, Representative population survey, Depression, Schizophrenia

## Abstract

Eine psychische Erkrankung bedeutet für viele Betroffene auch eine Auseinandersetzung mit den Reaktionen des Umfelds. Diese werden geprägt durch kulturell vorherrschende Vorstellungen von Ursache, Behandlung, Verlauf und biografischer Bedeutung der Krankheit. Der vorliegende Artikel gibt einen Überblick über die Entwicklung der Einstellungen der deutschen Bevölkerung zu psychischen Erkrankungen zwischen 1990 und 2020 mit Schwerpunkt auf Depression und Schizophrenie.

Mit Blick auf die letzten 30 Jahre zeigt sich, dass die Einstellungen zu psychischen Erkrankungen nicht statisch sind, sondern vielmehr einer Dynamik unterliegen, die sich in Abhängigkeit vom Krankheitsbild erheblich unterscheiden kann. Zusammenfassend ruft eine Depression weitaus weniger negative Gefühle hervor, als es bei der Schizophrenie der Fall ist. Dieser Unterschied hat sich in den letzten 30 Jahren vergrößert: Menschen mit Depressionen treffen heute auf mehr Verständnis als vor 30 Jahren, während die Stigmatisierung von Menschen mit Schizophrenie zugenommen hat. Neben einer zunehmenden Offenheit im Umgang mit psychischen Belastungen haben sich auch Normalitätsvorstellungen und Konzepte von psychischer Krankheit verändert. Depressive Zustandsbilder werden heute stärker mit dem eigenen Erleben in Verbindung gebracht als noch vor 10 Jahren. Eine Schizophrenie erscheint den Menschen dagegen heute eher fremdartiger. Während die Empfehlung sowohl von Psychotherapie als auch von Medikamenten für die Behandlung psychischer Krankheiten zunimmt und sowohl Psychotherapeuten als auch Psychiater häufiger als Anlaufstelle empfohlen werden, nimmt die Empfehlung spiritueller Helfer (Pfarrer, Priester) seit den 1990er-Jahren ab. Wir diskutieren mögliche Ursachen und Konsequenzen dieser divergenten Entwicklungen.

## Abstract (en)

For many of those affected, a mental illness also means dealing with the reactions of their environment. These are shaped by culturally prevailing ideas about the cause, treatment, course, and biographical significance of the illness. This article provides an overview of the development of population attitudes towards individuals with mental illness in Germany between 1990 and 2020 with a focus on depression and schizophrenia.

A look at the last 30 years shows that attitudes toward mental illness are not static; rather, they are subject to certain dynamics that can vary considerably depending on the type of mental illness. In summary, depression evokes far fewer negative emotions than is the case with schizophrenia. This gap in attitudes has widened over the last 30 years: people with depression are met with more understanding today than 30 years ago, while the stigma of schizophrenia seems to have increased. In addition to an increasing openness in dealing with mental stress, ideas of normality and concepts of mental illness seem to have also changed. Depressive states are more closely connected with people’s perceptions of their own experiences today than they were ten years ago. Schizophrenia, in turn, seems to be perceived as even more unfamiliar. While the recommendation of both psychotherapy and medication increases over time, and both psychotherapists and psychiatrists are more readily recommended as a source of help, recommendation of spiritual support (pastor, priest) declines steadily since 1990. We discuss potential causes and consequences of these divergent time trends.

## Der Einfluss der „externen Realität“ auf den Umgang mit psychisch erkrankten Menschen

Eine psychische Erkrankung bedeutet für die Betroffenen in der Regel nicht nur Herausforderungen und Beschränkungen durch die psychische Erkrankung selbst, sondern auch die Auseinandersetzung mit den Reaktionen des Umfelds. Angesichts der häufigen Erfahrung, gesellschaftlich benachteiligt und ausgegrenzt zu werden, spricht man auch von einer „zweiten Krankheit“, die zu den Symptomen und Belastungen der eigentlichen Krankheit hinzutritt. Dabei geht es nicht nur um Stigmatisierung, sondern auch um die kulturell vorherrschenden Vorstellungen von Ursachen, Behandlung, Verlauf und biografischer Bedeutung psychischer Krankheit. Wissen und Einstellungen zu psychischen Erkrankungen bilden einen kulturellen Kontext, der bestimmt, wie in einer Gesellschaft mit psychischen Krankheiten und den davon betroffenen Menschen umgegangen wird. Der amerikanische Soziologe Bruce Link nennt diesen kulturellen bzw. gesellschaftlich geteilten Kontext eine „externe Realität“ [1].

Die in der Bevölkerung verbreiteten Vorstellungen darüber, was psychische Krankheiten verursacht, wie sie behandelt werden sollten, welche Eigenschaften Menschen mit psychischen Krankheiten haben, welchen Verlauf psychische Krankheiten nehmen und schließlich welche Maßnahmen im Umgang mit psychischer Krankheit wirksam oder sinnvoll sind, beeinflussen, wie man sich selbst verhält, wenn man mit eigener oder fremder psychischer Krankheit konfrontiert wird. Wie groß der Einfluss der externen Realität aus Einstellungen und Haltungen auf das individuelle Handeln ist, wurde zum Beispiel während der COVID-19-Pandemie deutlich: Die Haltungen zum Impfen, zur Maskenpflicht und zu den Infektionsschutzmaßnahmen waren entscheidend für das individuelle Handeln und damit für die Wirksamkeit dieser Interventionen sowohl auf individueller Ebene als auch auf Bevölkerungsebene [2].

## Untersuchung der Veränderung von gesellschaftlichen Einstellungen

Dass Haltungen und Einstellungen der Allgemeinbevölkerung zu psychischen Krankheiten nicht statisch sind und sich zum Teil sogar erheblich verändern können, haben Trendstudien der letzten Jahre gezeigt. Für den deutschsprachigen Raum liefern die Daten aus der „Deutschen Langzeitstudie zum Stigma psychischer Krankheit“ [3] nicht nur aufgrund des Erhebungszeitraumes von 30 Jahren, sondern auch aufgrund der differenzierten Methodik, die in jeder Erhebung exakt repliziert wurde, eine weltweit einmalige Datenbasis, um Veränderungen in den gesellschaftlichen Einstellungen zu psychischen Erkrankungen über einen derart langen Zeitraum abzubilden. Die Studie wurde von Matthias Angermeyer in den 1990er-Jahren in mehreren bevölkerungsrepräsentativen Querschnittserhebungen begonnen und 2001 zum ersten Mal repliziert und seitdem in 2 weiteren Erhebungswellen 2011 und 2020 bis heute fortgeführt.

Grundlage dieser wiederholten Querschnittsstudien waren jeweils identische Fallbeschreibungen von je einer Person mit Depression und Schizophrenie sowie 1990 und 2011 zusätzlich von einer Person mit Alkoholabhängigkeit. Dabei wurden nur die Symptome und die aktuelle Situation beschrieben, aber keine Diagnose genannt. Dieses Vorgehen hat spezifische Vorteile im Vergleich zu Studien, die Krankheitsbezeichnungen („Depression“ „psychische Krankheit“ o. Ä.) verwenden. Indem detaillierte Fallbeschreibungen von spezifischen Krankheitsbildern als Stimulus verwendet werden, ist weitestgehend sichergestellt, dass der Stimulus, das geschilderte Verhalten, zu den verschiedenen Studienzeitpunkten auf sehr ähnliche Weise wahrgenommen wird und weniger einem Wandel von Begriffsbedeutungen unterliegt. Die Vorstellung davon, was eine „psychische Krankheit“ ist, mag sich im Lauf der Zeit verändern – die Schilderung der Symptome einer Schizophrenie z. B. wird dagegen auch nach 30 Jahren noch wortgleich gelesen und wahrscheinlich nahezu bedeutungsgleich verstanden. Alle Erhebungen der Langzeitstudie sind auch darüber hinaus methodisch sehr ähnlich, sowohl was die Stichprobengewinnung für die persönlichen Interviews angeht als auch hinsichtlich der Formulierung und Reihenfolge der Fragen und Antwortmöglichkeiten.

In diesem Artikel sollen vordergründig auf Grundlage der genannten deutschen Langzeitstudie, aber auch anhand von Untersuchungen aus anderen Ländern die wichtigsten Einstellungsentwicklungen seit 1990 nachgezeichnet werden [4–9]. Dabei fokussiert der Artikel auf Veränderungen von Normalitätsvorstellungen und Konzepten psychischer Gesundheit und Krankheit, auf die Entwicklung der Ablehnung von Menschen mit psychischen Krankheiten, auf Veränderungen der emotionalen Reaktionen sowie auf Veränderungen von Hilfesuchempfehlungen. Es werden mögliche Ursachen für divergente Einstellungsentwicklungen zu verschiedenen Diagnosen diskutiert und Impulse für mögliche Entstigmatisierungsstrategien gegeben.

## Wachsende Offenheit im Umgang mit psychischen Belastungen

Die psychische Gesundheit ist medial als Thema präsent. In der *New York Times* titelte ein Essay „We have reached peak ‚mental health‘“ [10] und brachte damit zum Ausdruck, dass die Berichterstattung und die Debatte über mentale Gesundheit einen Höhepunkt erreicht haben. Nicht zuletzt ist das Thema durch die Coronapandemie mit ihren zum Teil erheblichen Auswirkungen auf die psychische Gesundheit, insbesondere für jüngere Menschen [11], in den Fokus gerückt. Auch die psychiatrisch-psychotherapeutische Versorgung wird thematisiert: So gab es in Deutschland vieldiskutierte Fernsehsendungen zur Unterversorgung an Therapieplätzen [12] sowie Demonstrationen und Unterschriftensammlungen für mehr Therapieplätze (Deutsche Depressionsliga, „22 Wochen warten“). Die deutsche Psychotherapeutenvereinigung spricht im „Report Psychotherapie 2021“ davon, dass „auch aufgrund der insgesamt abnehmenden Stigmatisierung psychischer Krankheiten … die Inanspruchnahme von psychisch professioneller Hilfe“ angestiegen ist [13].

Auch quantitative Studien zeigen, dass psychische Belastungen inzwischen weniger tabuisiert werden. Ein Vergleich von jährlich wiederkehrenden Onlinebefragungen mit Quotenstichproben aus Irland konnte zum Beispiel zeigen, dass im Arbeitsalltag immer häufiger über psychische Probleme gesprochen wird: So stieg der Anteil der Personen, die berichteten, dass Kollegen oder Kolleginnen am Arbeitsplatz offen von eigenen psychischen Belastungen und Probleme berichtet hatten, von 14 % im Jahr 2019 auf 26 % im Jahr 2022 an [14]. Die Evaluation der britischen Time-to-Change-Kampagne gegen Stigmatisierung (2008–2019) konnte eindrucksvoll zeigen, dass immer mehr Menschen in der Allgemeinbevölkerung angaben, Kontakt zu einem Menschen mit einer psychischen Krankheit gehabt zu haben [15].

Auch stigmatisierende Einstellungen gegenüber Menschen mit psychischen Krankheiten gingen im Vereinigten Königreich im Evaluationszeitraum der Kampagne zurück. Diese Veränderung zeigte sich in Reaktion auf Begriffe wie „psychische Krankheit“ oder „mentale Gesundheitsprobleme“. Gleichzeitig zeigte sich aber auch eine Verbreiterung bzw. Normalisierung des Konzepts von psychischer Krankheit: Immer häufiger bejahten die Befragten im Verlauf der Kampagne die Frage, ob etwa Trauer oder Stress eine psychische Krankheit seien [15]. So stieg der Anteil derjenigen, die Stress als „psychische Krankheit“ einordneten, von 57,5 % im Jahr 2009 auf 66,2 % im Jahr 2017 [16]. Offensichtlich haben sich in den letzten Jahrzehnten nicht nur die Einstellungen zu psychischen Problemen oder Krankheiten verändert, sondern auch die Vorstellungen davon, welche Zustände als psychische Krankheit beschrieben werden können. Vermutlich werden zunehmend auch leichte oder mittelgradige Beschwerdebilder dieser Kategorie zugeordnet, was die Bedeutung der beobachteten Abnahme des Stigmas relativiert: Wenn weniger schwere Krankheitsbilder mit dem Begriff „psychische Krankheit“ assoziiert werden, bezieht sich auch die wachsende Toleranz auf weniger schwerwiegende Krankheitszustände.

## Veränderte Normalitätsvorstellungen und Konzepte von psychischer Krankheit

**Figure 1:**
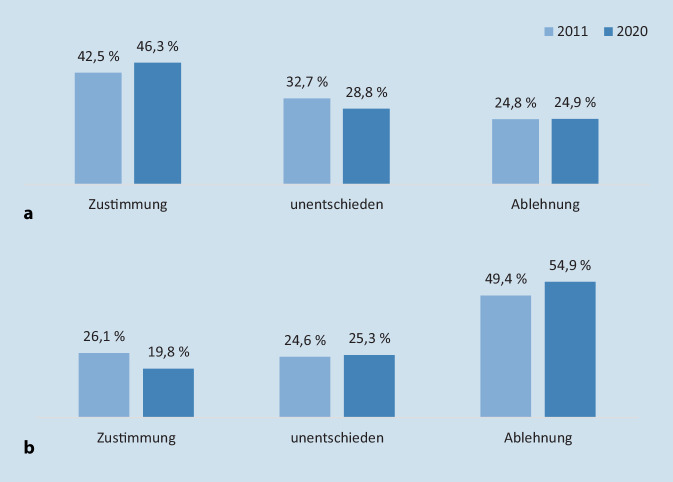

Wenn offener über psychische Belastungen gesprochen wird und Menschen häufiger mit Menschen ins Gespräch kommen, die an psychischen Erkrankungen leiden oder gelitten haben, dann verändern sich vermutlich auch die Vorstellungen davon, was „normal“ ist und was eine psychische Krankheit ausmacht. In der deutschen Langzeitstudie wurde z. B. in Bezug auf die geschilderten Symptome zur Depression die Einstellung zu folgender Aussage erfragt: „Im Grunde geht es uns allen manchmal so wie dieser Person. Es ist nur eine Frage, wie ausgeprägt der Zustand ist.“ Hierbei zeigte sich, dass sich die Einschätzung, ob die Symptome auf einem Kontinuum mit dem eigenen Erleben der Respondenten lokalisiert werden können, zwischen 2011 und 2020 deutlich verändert hat. Ein Kontinuum-Modell psychischer Krankheit geht davon aus, dass es keinen „Schwarz-Weiß“-Unterschied zwischen psychischer Gesundheit und Krankheit gibt, sondern dass es einen fließenden Übergang zwischen einem Zustand vollkommener psychischer Gesundheit und einem Zustand schwerster psychischer Krankheit gibt. Die Langzeitstudie zeigt nun, dass das geschilderte Verhalten den Menschen in Deutschland zunehmend näher gerückt zu sein scheint und stärker mit dem eigenen Erleben in Verbindung gebracht wird: Während 2011 noch 42,5 % zustimmten, dass die Symptome einer Depression auf einem Kontinuum mit eigenen Erfahrungen liegen, waren es 2020 bereits 46,3 % (Abb. 1). Im Jahr 2011 lehnten es 44,1 % der Befragten ab, das Verhalten als „fremdartig“ einzuschätzen, 2020 waren es schon 53,8 % [8]. Bei der Schizophrenie zeigt sich hingegen eine Abnahme von Kontinuumsvorstellungen. Diese wurden im Jahr 2011 nur von 26,1 % der Respondenten in Bezug auf das Beschwerdebild einer Schizophrenie bejaht. 9 Jahre später, im Jahr 2020, hatte die Zustimmung weiter auf 19,8 % abgenommen (Abb. 1).

Depressionen treten nicht nur häufiger auf als Schizophrenien, es wird auch wesentlich häufiger über sie berichtet [17]. Während die Berichterstattung über die Schizophrenie in den Printmedien vor allem auf Verbrechen fokussiert und kaum Therapiemöglichkeiten erwähnt, zeichnen diese Medien ein wesentlich ausgewogeneres Bild von der Depression [17]. Unterschiedliche Wahrnehmungen der Krankheitsbilder werden also zumindest durch die Printmedien noch verstärkt.

## Ruf nach mehr professionellen therapeutischen Behandlungen

In den letzten 30 Jahren ist der Ruf nach professionellen therapeutischen Behandlungen von psychischen Störungen lauter geworden. Diese wurden in der deutschen Langzeitstudie als Empfehlung für die in der Fallgeschichte geschilderte Person erfragt – jede einzelne vorgeschlagene Maßnahme wurde von den Befragten mittels einer 5‑Punkt-Likert-Skala von „würde ich dringend empfehlen“ bis „würde ich dringend davon abraten“ bewertet, die beiden affirmativen Kategorien werden hier als „Empfehlung“ zusammengefasst. Von Beginn an wurde in der Studie die Behandlung mittels Psychotherapie sowohl für die Schizophrenie als auch für die Depression von mehr Befragten präferiert als die Behandlung mittels Medikamente [18]. Analog verhält es sich für die Empfehlung von Psychotherapeuten bzw. Psychiatern^1^: Während in den 1990er-Jahren über die Hälfte der Respondenten einer an Depression (57 %) oder Schizophrenie (65 %) erkrankten Person den Besuch eines Psychotherapeuten empfahlen, erhöhte sich dieser Anteil bis zum Jahr 2020 für beide Krankheitsbilder um mehr als 20 Prozentpunkte (Depression: 80 %, Schizophrenie: 89 %). Auch die Empfehlungsraten für einen Psychiater stiegen im Zeitraum für das Beschwerdebild einer Depression von 54 % auf 65 % bzw. für das Beschwerdebild einer Schizophrenie von 65 % auf 84 % im Jahr an. Dies entspricht einem Zuwachs von 11 (Depression) bzw. 19 Prozentpunkten (Schizophrenie; [19]).

Weitestgehend unverändert blieben hingegen die Empfehlungen der Behandlung bei einem Hausarzt. Für das Beschwerdebild einer Depression lag die Empfehlungsrate hier im Jahr 1990 bei 74 % und für das einer Schizophrenie bei 69 %. Im Jahr 2020 waren es für beide Krankheitsbilder 74 %. Zusätzlich zu den therapeutischen Berufsgruppen wurden den Teilnehmenden auch Seelsorger als potenzielle Hilfsangebote vorgeschlagen. Hier zeichneten sich jedoch für Priester und Pfarrer insgesamt deutlich niedrigere Empfehlungsraten ab. Vor allem zeigte sich im Zeitverlauf ein deutlicher Rückgang: Bereits im Jahr 1990 wurden Priester und Pfarrer nur von einer Minderheit der Befragten für die Behandlung einer Depression (28 %) oder einer Schizophrenie (25 %) empfohlen. Im Jahr 2020 waren die Empfehlungsraten für beide Beschwerdebilder auf jeweils 15 % gesunken [19].

Zusammenfassend werden bei der Behandlung einer psychischen Erkrankung, wie der einer Depression oder einer Schizophrenie, professionelle Behandler mit einer „Psycho“-Spezialisierung im Vergleich zu anderen Berufsgruppen klar bevorzugt empfohlen. Dabei werden Psychotherapeuten häufiger empfohlen als Psychiater. An zweiter Stelle folgen die Hausärzte, während andere Helfer etwa aus dem Bereich der Seelsorge an Popularität verlieren. Diese Veränderungen können einerseits auf eine gestiegene Gesundheitskompetenz bzgl. psychischer Gesundheit (Mental Health Literacy) zurückgeführt werden und als Beleg dafür dienen, dass die in den Fallvignetten geschilderten akuten Krankheitsbilder stärker als solche erkannt und weniger als Lebenskrise verstanden werden. Auf der anderen Seite zeigen die Ergebnisse aber auch einen Bedeutungsverlust (nicht therapeutisch arbeitender) kirchlicher Berufsgruppen, die bei der Bewältigung von Lebenskrisen offenbar eine immer geringere Bedeutung haben [19]. Der Bedeutungsverlust alternativer Helfersysteme trägt möglicherweise auch zur aktuellen Überlastung insbesondere des Systems der Psychotherapie bei.

## Präferenzen für die Ressourcenallokation im Gesundheitswesen

Seit 2001 wurde im Rahmen der deutschen Langzeitstudie auch der Frage nachgegangen, in welchen Bereichen der Gesundheitsversorgung Kosteneinsparungen aus Sicht der Studienteilnehmer und Studienteilnehmerinnen am ehesten denkbar wären bzw. in welchen Bereichen auf keinen Fall gespart werden sollte. Zur Wahl standen insgesamt 9 körperliche und psychische Krankheiten, von denen 3 Krankheitsbilder ausgewählt werden sollten, bei denen trotz finanzieller Knappheit auf keinen Fall Mittel gekürzt werden sollten.

Im Jahr 2001 standen somatische Krankheitsbilder noch eindeutig an der Spitze der Liste, angeführt von Krebserkrankungen, bei denen über 75 % der Teilnehmenden Kürzungen ablehnten, gefolgt von Herz-Kreislauf-Erkrankungen oder Aids, die jeweils von knapp der Hälfte der Respondenten ausgewählt worden. Schizophrenie, Depression und Alkoholabhängigkeit lagen hingegen auf den 3 letzten Plätzen. 2011 hatte sich dieses Bild nur leicht verändert: Während Schizophrenie und Alkoholabhängigkeit nach wie vor am Ende der Liste auftauchten und jeweils von weniger als 10 % der Befragten priorisiert wurden, lag die Depression mit etwa 18 % bereits an sechster Stelle. Unter den Eindrücken der Pandemie und der tatsächlichen Priorisierung von bestimmten Gesundheitsleistungen im Gesundheitssystem, war die Depression im Jahr 2020 auf Rang 4 der Liste aufgestiegen. Jeder vierte Respondent gab an, dass die Mittel für die Behandlung von Menschen mit Depressionen auf keinen Fall gekürzt werden sollten. Auf den beiden letzten Plätzen lagen dagegen unverändert die Beschwerdebilder Schizophrenie und Alkoholabhängigkeit [20].

Bei der Ressourcenverteilung im Gesundheitswesen deutet sich hier eine Zweiteilung an: Während die Depression als Krankheit offensichtlich an Wichtigkeit gewonnen hat, scheinen die Ressourcen für Menschen mit anderen, in der Regel schweren psychischen Krankheiten oder Suchtkrankheiten nach wie vor niedrige Priorität in der Allgemeinbevölkerung zu haben. Auch Befragungen von Studierenden unterschiedlicher Fachrichtungen ergaben, dass Suchterkrankungen im Vergleich zu anderen psychischen und somatischen Erkrankungen als weniger schwerwiegend und verstärkt selbstverschuldet eingeschätzt wurden und am ehesten als Erkrankung mit finanziellen Einsparpotenzialen in der Gesundheitsversorgung gewählt wurden [21].

## Eine zweigeteilte Entwicklung der gesellschaftlichen Akzeptanz?

Dafür, dass Menschen mit einer Suchterkrankung oder einer Schizophrenie in besonderem Maße stigmatisiert werden, sprechen auch die empirischen Befunde zur „sozialen Distanz“. Die soziale Distanz ist ein in der psychiatrischen Einstellungsforschung häufig verwendetes Maß für die Diskriminierung von Menschen mit psychischen Krankheiten. Gemessen wird hierbei die Bereitschaft, mit einer psychisch erkrankten Person in verschiedenen Alltagssituationen in Kontakt zu treten. In der deutschen Langzeitstudie zeigt sich, dass diese Bereitschaft in den Jahren von 1990 bis 2011 insbesondere für das Beschwerdebild einer Schizophrenie deutlich abgenommen hat, ein Hinweis auf eine Zunahme von Stigmatisierung. Für das Beschwerdebild einer Depression zeigten sich hingegen keine einheitlichen Veränderungen.

Eine aktuelle Trendstudie aus den Vereinigten Staaten [22] zeigt nun, dass auch dort die Ablehnung von an Schizophrenie erkrankten Menschen eher zunimmt. Auffällig war jedoch auch ein starker Abfall des Bedürfnisses nach sozialer Distanz gegenüber Menschen mit Depression zwischen 2006 und 2018. Im Jahr 2018 lag hier die Ablehnung der Person nur unwesentlich über der Ablehnung einer Person, die Alltagsprobleme, aber keine Anzeichen einer psychischen Krankheit aufwies. In der deutschen Studie zeigten sich leichte Verbesserungen in den Einstellungen zur Depression insbesondere nach 2001, während die Verschlechterungen bei der Schizophrenie vor allem zwischen 1990 und 2001 beobachtet wurden [9].

Insgesamt zeigen sowohl die deutschen als auch die amerikanischen Daten unterschiedliche Entwicklungen für die Krankheitsbilder Schizophrenie und Depression: Während die Depression offensichtlich dem eigenen Erleben der Respondenten näher gerückt zu sein scheint und als weniger fremdartig wahrgenommen wird, wird die Schizophrenie nach wie vor und zunehmend als außerhalb des eigenen Erlebens wahrgenommen und mit einem stärkeren Bedürfnis nach sozialer Distanz in Verbindung gebracht. In dieses Bild passt, dass in den Vereinigten Staaten auch die Einschätzung der Eigengefährdung der betroffenen Person und der von ihr ausgehenden Fremdgefährdung für die Schizophrenie zwischen 1996 und 2018 signifikant angestiegen ist ebenso die Zustimmung zur Krankenhausbehandlung gegen den Willen der Person [23]. Für die Depression fanden sich nur Veränderungen hinsichtlich der Eigengefährdung, nicht aber hinsichtlich Fremdgefährdung und der Präferenz für Zwangsmaßnahmen.

## Mögliche Ursachen für divergente Einstellungsentwicklungen

Auf Basis der vorgestellten Befunde erscheint ein allgemeines Resümee, wie sich die Einstellungen zu psychischen Erkrankungen in Deutschland entwickelt haben, auf den ersten Blick schwierig. In Abhängigkeit vom Krankheitsbild gibt es unterschiedliche Verläufe: Eine Depression scheint heutzutage weitaus weniger negative Gefühle hervorzurufen als eine Schizophrenie oder eine Suchterkrankung. Auch scheinen Menschen mit Depressionen heute auf mehr Verständnis zu treffen als noch vor 30 Jahren. Im Gegensatz dazu ruft eine Schizophrenie heutzutage sogar noch mehr Ängste hervor, als es vor 30 Jahren noch der Fall war, Suchterkrankungen werden nach wie vor am stärksten stigmatisiert.

Das eingangs bereits zitierte Essay in der *New York Times* über den „peak mental health“ [10] macht den öffentlichen Diskurs über psychische Gesundheit für eine solche Entwicklung verantwortlich. Der Autor kritisiert, dass das häufige Sprechen über psychische Gesundheit zwar die psychischen Belastungen des Einzelnen kommunizierbar macht, aber damit die Aufmerksamkeit von schweren psychischen Krankheiten und den davon Betroffenen ablenkt: „[Die Verwendung des Begriffs] ‚Psychische Gesundheit‘ beschwört Phänomene herauf, die mehr oder weniger nachvollziehbar sind: Ängste und Depressionen. Aber wer wird dadurch ausgeschlossen? Die veränderte Sprechweise sollte Stigma reduzieren. Aber stattdessen hat sie unsere Aufmerksamkeit genau von den Menschen abgelenkt, die am stärksten stigmatisiert werden – z. B. von denjenigen mit der Diagnose Schizophrenie.“

Ein allgemeiner Diskurs über Überlastung, das Aufrechterhalten einer „Work-Life-Balance“ und die Sorge um die eigene psychische Gesundheit, der zunächst eine wachsende gesellschaftliche Akzeptanz seelischer Leiden signalisiert, führt also nicht notwendigerweise auch dazu, dass Menschen mit schweren psychischen Krankheiten entstigmatisiert werden. Im Gegenteil, es ist vorstellbar, dass der Fokus auf (eigene) Belastung und Überlastung paradoxerweise dazu führt, dass Menschen mit schweren, oft auch anstrengenden und das Umfeld belastenden psychischen Krankheiten aus dem Blickfeld geraten und in der Folge umso stärker abgelehnt werden. Der Zusammenhang zwischen subjektiver Überlastung und der Ablehnung von Menschen mit schweren psychischen Krankheiten ist allerdings bis jetzt nicht empirisch belegt.

Eine andere mögliche Erklärung insbesondere für die Zunahme der Ablehnung von Menschen mit Schizophrenie liegt in der zunehmenden Biologisierung des Krankheitsverständnisses der Öffentlichkeit, insbesondere für dieses Krankheitsbild. Eine Metaanalyse aus dem Jahr 2012 [24] konnte zeigen, dass im Verlauf der 1990er-Jahre die Zustimmung zu biologischen oder genetischen Krankheitsursachen sowohl für die Depression als auch für die Schizophrenie deutlich angestiegen ist, bei der Schizophrenie aber insgesamt wesentlich höher lag als bei der Depression. Zahlreiche experimentelle und korrelative Studien haben mittlerweile gezeigt, dass Vorstellungen von genetischen Krankheitsursachen mit einer stärkeren Ablehnung der davon betroffenen Menschen verbunden sind [7, 25, 26]. Durch die Annahme biologischer Krankheitsprozesse wird offenbar die wahrgenommene Andersartigkeit der Person verstärkt sowie die Möglichkeit einer erfolgreichen Therapie geringer eingeschätzt.

Unklar ist, inwieweit die Mediendarstellung von psychischen Krankheiten zur divergenten Einstellungsentwicklung beiträgt. Wie bereits beschrieben wird tatsächlich sehr unterschiedlich über Depressionen und Schizophrenie berichtet [17] und die Berichterstattung über Schizophrenie (und über Suchtkrankheiten) entspricht den empirisch gefundenen negativen Einstellungen der Allgemeinbevölkerung. Allerdings sind die Medien vermutlich gleichzeitig Ursache und Folge bestimmter kultureller Konzeptionen von psychischer Krankheit. Während sich in den 1990er-Jahren ein zeitlicher Zusammenhang zwischen Medienberichterstattung über Gewalttaten in Verbindung mit psychischer Krankheit und einer Zunahme der Ablehnung zeigte [27], fand sich dieser Zusammenhang in den 2010er-Jahren nur wesentlich schwächer [28–30].

## Fazit

Der kulturelle Kontext, in dem psychische Krankheit heute erlebt wird, hat sich in den letzten Jahren deutlich gewandelt. Zusammenfassend kann man schlussfolgern, dass psychische Belastung, psychische Probleme und leichte bis mittelschwere psychische Krankheiten heute leichter kommunizierbar sind als noch vor 20 oder 30 Jahren. Auch die Inanspruchnahme von Hilfen für diese Störungen scheint zuzunehmen, wie sich auch an den steigenden Bedarfen für Psychotherapie zeigt [13]. Auf der anderen Seite haben sich die Einstellungen gegenüber Menschen mit Suchtkrankheiten und Menschen mit Schizophrenie nicht verbessert, zum Teil sogar verschlechtert. Hier gibt es Gruppen von Patienten und Patientinnen, die nicht im Fokus der öffentlichen Aufmerksamkeit stehen und wenn, dann in einem negativen, stigmatisierenden Kontext. Um den Umgang mit diesen Krankheitsbildern im Sinne einer niederschwelligeren Inanspruchnahme von Hilfen, früherer Interventionen und mehr Teilhabe zu verwirklichen, müssen sich Entstigmatisierungsbemühungen auf diese Krankheiten fokussieren.

Die unterschiedlichen Einstellungsdynamiken der letzten Jahre zeigen auch, dass Maßnahmen zur Stärkung der gesellschaftlichen Akzeptanz von Menschen mit psychischen Krankheiten an der tatsächlichen Einstellungsentwicklung ausgerichtet und anhand dieser überprüft werden müssen. Eine Normalisierung von psychischen Krankheiten allgemein ist offenbar nicht ausreichend. Ein Übergreifen von positiven Veränderungen auf schwerere Krankheitsbilder lässt sich anhand der bisher vorliegenden Daten jedenfalls nicht belegen. Anti-Stigma-Aktivitäten müssen maßgeblich von Menschen mit eigener Krankheitserfahrung gestaltet und geleitet werden, ein herausragendes Beispiel ist dabei die Anti-Stigma-Kampagne MV der Sozialpsychiatrie Mecklenburg-Vorpommern, die in einem trialogischen Format (Betroffene, Angehörige, Profis) sehr überzeugende, selbstbewusste und charmant selbstironische Plakatmotive entwickelte und realisierte [31]. Kontaktbasierte, gezielte, kontextspezifische Interventionen auch für Suchtkrankheiten und psychotische Störungen sind nach wie vor die am besten belegte Strategie, um Stigmatisierung zu reduzieren [32]. Um Kontakt zu ermöglichen, muss gesellschaftlich und im sozialen Nahraum ein Umfeld geschaffen werden, das es Menschen leichter macht, über ihre Krankheitserfahrungen zu sprechen.

## Footnotes

1 Da in der Langzeitstudie seit 1990 das generische Maskulinum verwendet wird und der Wortlaut aus methodischen Gründen nicht angepasst werden sollte, berichten wir hier ebenfalls im generischen Maskulinum.

## References

[1] Link BG (2013). It is time to change our cultural context. Invited commentary on...Evaluation of England’s Time to Change programme. Br J Psychiatry Suppl.

[2] Nivette A, Ribeaud D, Murray A, Steinhoff A, Bechtiger L, Hepp U, Shanahan L, Eisner M (2021). Non-compliance with COVID-19-related public health measures among young adults in Switzerland: insights from a longitudinal cohort study. Soc Sci Med.

[3] Angermeyer MC, Matschinger H (1996). The effect of personal experience with mental illness on the attitude towards individuals suffering from mental disorders. Soc Psychiatry Psychiatr Epidemiol.

[4] Angermeyer MC, Matschinger H, Siara CE (1992). Wissensbestände, Überzeugungssysteme und Einstellungsmuster der Bevölkerung der Bunderepublik Deutschland bezüglich psychischer Erkrankungen. Abschlussbericht.

[5] Angermeyer MC, Beck M, Matschinger H (2003). Determinants of the public’s preference for social distance from people with schizophrenia. Can J Psychiatry.

[6] Angermeyer MC, Matschinger H, Schomerus G (2013). Attitudes towards psychiatric treatment and people with mental illness: changes over two decades. Br J Psychiatry.

[7] Schomerus G, Matschinger H, Angermeyer MC (2014). Causal beliefs of the public and social acceptance of persons with mental illness: a comparative analysis of schizophrenia, depression and alcohol dependence. Psychol Med.

[8] Schomerus G, Schindler S, Baumann E, Angermeyer MC (2022). Changes in continuum beliefs for depression and schizophrenia in the general population 2011–2020: a widening gap. Soc Psychiatry Psychiatr Epidemiol.

[9] Schomerus G, Schindler S, Sander C, Baumann E, Angermeyer MC (2022). Changes in mental illness stigma over 30 years—improvement, persistence, or deterioration?. Eur Psychiatr.

[10] Green H (2022) We have reached peak ‘mental health’. The New York Times. https://www.nytimes.com/2022/09/20/opinion/us-mental-health-awareness.html. Zugegriffen: 21. Okt. 2022

[11] Dogan-Sander E, Kohls E, Baldofski S, Rummel-Kluge C (2021). More depressive symptoms, alcohol and drug consumption: increase in mental health symptoms among university students after one year of the COVID-19 pandemic. Front Psychiatry.

[12] ZDF Magazin Royale (2022) Warten, warten, warten. Psychotherapie. YouTube. https://www.google.com/url?sa=t&rct=j&q=&esrc=s&source=web&cd=&cad=rja&uact=8&ved=2ahUKEwj4lNv85sb6AhUGQ_EDHfs-DHUQwqsBegQIBxAB&url=https%3A%2F%2Fwww.youtube.com%2Fwatch%3Fv%3DmzMj-v1sMI4&usg=AOvVaw32dt9vRK6vfQjr-akNIzmg. Zugegriffen: 21. Okt. 2022

[13] Deutsche PsychotherapeutenVereinigung e. V. (2021) Report Psychotherapie 2021. https://www.dptv.de/fileadmin/Redaktion/Bilder_und_Dokumente/Wissensdatenbank_oeffentlich/Report_Psychotherapie/DPtV_Report_Psychotherapie_2021.pdf. Zugegriffen: 21. Okt. 2022

[14] St Patrick’s Mental Health Services (2022) Annual Attitudes to Mental Health and Stigma Survey: Five-year (2018–2022) comparative review. https://www.stpatricks.ie/media/3521/2022-annual-stigma-and-attitudes-to-mental-health-survey.pdf. Zugegriffen: 21. Okt. 2022

[15] Robinson EJ, Henderson C (2017). Public knowledge, attitudes, social distance and reporting contact with people with mental illness 2009–2017. Psychol Med.

[16] Robinson EJ, Henderson C (2017). Public knowledge, attitudes, social distance and reporting contact with people with mental illness 2009–2017. Supplementary material. Psychol Med.

[17] Rosset M, Freytag A, Dittrich A, Jaspersen M, Baumann E (2020). Psychische Erkrankungen in Medienberichten. Befunde zur Darstellung und Wahrnehmung. Commun Social.

[18] Angermeyer MC, van der Auwera S, Carta MG, Schomerus G (2017). Public attitudes towards psychiatry and psychiatric treatment at the beginning of the 21st century: a systematic review and meta-analysis of population surveys. World Psychiatry.

[19] Angermeyer MC, Schindler S, Matschinger H, Baumann E, Schomerus G The rise in acceptance of mental health professionals: Help-seeking recommendations of the German public 1990–2020. Epidemiol Psychiatr Sci (in press). 10.1017/S204579602300001X 10.1017/S204579602300001X PMC9971855 36786061

[20] Schomerus G, Baumann E, Sander C, Speerforck S, Angermeyer MC (2021). Some good news for psychiatry: resource allocation preferences of the public during the COVID-19 pandemic. World Psychiatry.

[21] Schmidt H, Koschinowski J, Bischof G, Schomerus G, Borgwardt S, Rumpf H-J (2022). Einstellungen von Medizinstudierenden gegenüber alkoholbezogenen Störungen: Abhängig von der angestrebten medizinischen Fachrichtung?. Psychiatr Prax.

[22] Pescosolido BA, Halpern-Manners A, Luo L, Perry B (2021). Trends in public stigma of mental illness in the US, 1996–2018. JAMA Netw Open.

[23] Pescosolido BA, Manago B, Monahan J (2019). Evolving public views on the likelihood of violence from people with mental illness: stigma and its consequences. Health Aff.

[24] Schomerus G, Schwahn C, Holzinger A, Corrigan PW, Grabe HJ, Carta MG, Angermeyer MC (2012). Evolution of public attitudes about mental illness: a systematic review and meta-analysis. Acta Psychiatr Scand.

[25] Kvaale EP, Gottdiener WH, Haslam N (2013). Biogenetic explanations and stigma: a meta-analytic review of associations among laypeople. Soc Sci Med.

[26] Kvaale EP, Haslam N, Gottdiener WH (2013). The ‘side effects’ of medicalization: a meta-analytic review of how biogenetic explanations affect stigma. Clin Psychol Rev.

[27] Angermeyer MC, Matschinger H (1995). Violent attacks on public figures by persons suffering from psychiatric disorders. Their effect on the social distance towards the mentally ill. Eur Arch Psychiatry Clin Neurosci.

[28] von dem Knesebeck O, Mnich E, Angermeyer MC, Kofahl C, Makowski A (2015). Changes in depression stigma after the Germanwings crash—Findings from German population surveys. J Affect Disord.

[29] Schomerus G, Stolzenburg S, Bauch A, Speerforck S, Janowitz D, Angermeyer MC (2017). Shifting blame? Impact of reports of violence and mental illness in the context of terrorism on population attitudes towards persons with mental illness in Germany. Psychiatry Res.

[30] Schomerus G, Stolzenburg S, Angermeyer MC (2015). Impact of the Germanwings plane crash on mental illness stigma: results from two population surveys in Germany before and after the incident. World Psychiatry.

[31] Landesverband Sozialpsychiatrie M‑V e. V. (2016) Anti Stigma Kampagne M‑V. http://antistigma-mv.de/. Zugegriffen: 21. Okt. 2022

[32] Corrigan PW (2011). Strategic stigma change (SSC): five principles for social marketing campaigns to reduce stigma. PS.

